# Plant mineral nutrition and disease resistance: A significant linkage for sustainable crop protection

**DOI:** 10.3389/fpls.2022.883970

**Published:** 2022-10-20

**Authors:** Ruchi Tripathi, Rashmi Tewari, K. P. Singh, Chetan Keswani, Tatiana Minkina, Anoop Kumar Srivastava, Ugo De Corato, Estibaliz Sansinenea

**Affiliations:** ^1^Department of Plant Pathology, College of Agriculture, G. B. Pant University of Agriculture and Technology, Pantnagar, India; ^2^Academy of Biology and Biotechnology, Southern Federal University, Rostov-on-Don, Russia; ^3^ICAR – Central Citrus Research Institute, Nagpur, Nagpur, Maharashtra, India; ^4^Division of Bioenergy, Biorefinery and Green Chemistry (BBC-BIC), Department of Energy Technologies and Renewable Resources (TERIN), Italian National Agency for New Technologies, Energy and Sustainable Economic Development (ENEA), Bari, Italy; ^5^Faculty of Chemical Sciences, Benemerita, Autonomous University of Puebla, Puebla, Mexico

**Keywords:** mineral nutrition, disease management, plant growth, nutrient signaling, nutrient use efficiency

## Abstract

Complete and balanced nutrition has always been the first line of plant defense due to the direct involvement of mineral elements in plant protection. Mineral elements affect plant health directly by modulating the activity of redox enzymes or improving the plant vigor indirectly by altering root exudates, and changing microflora population dynamics, rhizosphere soil nutrient content, pH fluctuation, lignin deposition, and phytoalexin biosynthesis. Nitrogen (N) is one of the most important macronutrients having a significant impact on the host-pathogen axis. N negatively affects the plant’s physical defense along with the production of antimicrobial compounds, but it significantly alleviates defense-related enzyme levels that can eventually assist in systemic resistance. Potassium (K) is an essential plant nutrient, when it is present in adequate concentration, it can certainly increase the plant’s polyphenolic concentrations, which play a critical role in the defense mechanism. Although no distinguished role of phosphorus (P) is observed in plant disease resistance, a high P content may increase the plant’s susceptibility toward the invader. Manganese (Mn) is one of the most important micronutrients, which have a vital effect on photosynthesis, lignin biosynthesis, and other plant metabolic functions. Zinc (Zn) is a part of enzymes that are involved in auxin synthesis, infectivity, phytotoxin, and mycotoxin production in pathogenic microorganisms. Similarly, many other nutrients also have variable effects on enhancing or decreasing the host susceptibility toward disease onset and progression, thereby making integrative plant nutrition an indispensable component of sustainable agriculture. However, there are still many factors influencing the triple interaction of host-pathogen-mineral elements, which are not yet unraveled. Thereby, the present review has summarized the recent progress regarding the use of macro- and micronutrients in sustainable agriculture and their role in plant disease resistance.

## Introduction

**FIGURE 1:**
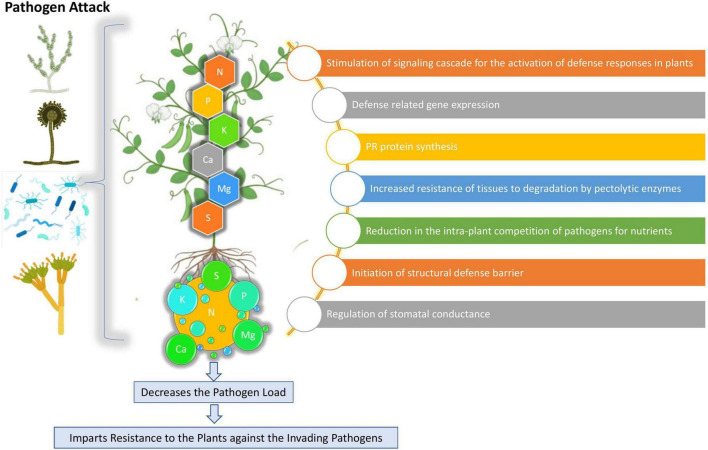
Overview of the biochemical and physiological roles of macronutrients.

**FIGURE 2:**
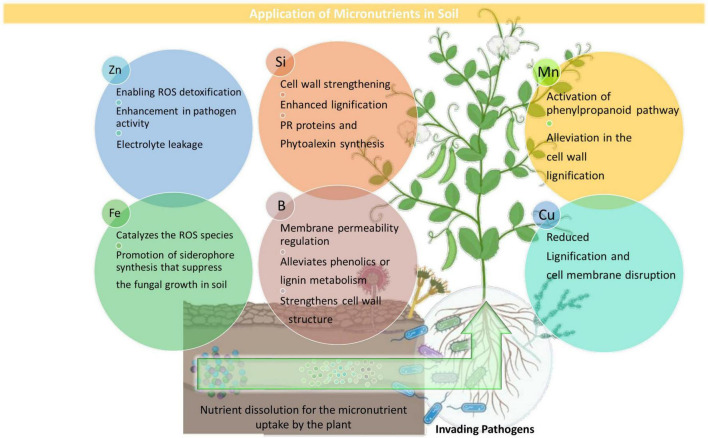
Overview of the biochemical and physiological roles of major micronutrients.

Crop production remains delimited by an array of biotic and abiotic factors that can eventually reduce crop yield, quantity, and quality (Wang M. et al., 2013). Among the biotic factors, phytopathogens such as bacteria, fungi, nematodes, and viruses, have considerable impacts on agricultural productivity and sustainability. Sustainable agriculture can be said as the utilization of the agricultural ecosystem in a way that enables the perfect balance of biological diversity, productivity, and regeneration capacity so that the present and significant future demands can be fulfilled without harming other ecosystems (Lewandowski et al., 1999) and at the same time by managing plant diseases along with an increased yield and improved product quality (Camprubi et al., 2007). Developing along with evolution course, plants have developed multi-layered defense systems enabling them to resist and/or tolerate pathogen invasion and resist infection (Sun et al., 2020). The mineral nutrients play a potential role in supporting plant wellness that is influenced by various abiotic factors, such as light, humidity, temperature, and mineral nutrients (Velasquez et al., 2018) The N status can be affected by high soil temperature as it increases the overall N availability in soils (Lukac et al., 2011) and also increases the plant metabolic rate, thereby positively correlating N uptake with temperature (Dong et al., 2001). The K demands are observed to increase under low moisture conditions, which may sequester a higher reactive oxygen species (ROS) production leading to increased disruption of the plant cell organelles (Wang Y. et al., 2013). The P availability was reported to decrease during high light intensity, which subsequently increased the root length and fine root hair production (Wen et al., 2017). Mineral nutrients are particularly and directly involved in plant protection as structural components and metabolic regulators (Huber, 1980). As imparting the primary defense line, the plant’s nutritional status can play a deciding role in determining the plants’ susceptibility or resistance toward the invading pathogens (Walters and Bingham, 2007; Marschner and Marschner, 2012). The mineral elements can potentially influence plant health either directly by activating the enzymes involved in the synthesis of defense metabolites (callose, glucosinolates, lignin, phenols, and phytoalexins) or indirectly by altering the microbial activity, root exudates composition, and rhizosphere pH modulation (Datnoff et al., 2007). For controlling and managing plant disease, balanced nutrition had always been the primary component, yet its importance remains to be unraveled. The importance of mineral nutrition on plant disease management can be highlighted as (a) fertilization effect on the incidence or severity of a particular pathogen/host pathosystem, (b) mineral nutrition effect in imparting resistance or susceptibility to plant when provided in different concentration, and (c) effect of specific nutrient availability or starvation on disease in consortium with the growth stage of the plant, environmental conditions, and biological activity, which can eventually affect the outcomes (Meena et al., 2015). A healthy plant will certainly have high vigor and improved resistance and hereby mineral nutrients show their capabilities in disease management (Ojha and Jha, 2021). Mineral nutrients, such as the primary macronutrients, nitrogen (N), phosphorus (P), and potassium (K); the three secondary macronutrients, calcium (Ca), sulfur (S), and magnesium (Mg); and the micronutrients, boron (B), manganese (Mn), iron (Fe), zinc (Zn), copper (Cu), and silicon (Si), are significant in imparting disease resistance and healthy growth to the plant (Datnoff et al., 2007; Gupta et al., 2017) (Figures 1, 2). Some key mineral elements have a greater impact on plant disease, for instance, N, which can limit the pathogen growth and may also affect the plant defense elicitation and deployment. Moreover, the availability of different N forms (NH_4_^+^ and NO_3_^–^) also shows varied effects on plant disease resistance using the assimilatory and metabolic pathways (Bolton and Thomma, 2008; Mur et al., 2017). Similarly, K is particularly a critical element required for plant growth and metabolism and contributes greatly to plants’ survival under various biotic stresses (Pettigrew, 2008) by assisting them in multiple plant defense enzyme functioning, regulating the higher plants’ metabolite patterns, and eventually altering the metabolite concentrations (Mengel, 2001). It can be noted that a particular nutrient may have opposite impacts on different diseases and in different environments, i.e., the same nutrient may increase the incidence of one disease but at the same time may decrease the incidence of others (Agrios, 2005) (Table 1).

**TABLE 1:** Effect of major plant nutrients on disease reactions.

Mineral element	Crop	Disease	Causal organism	Effect on disease reaction	References
**Macronutrients**					
Nitrogen	Tomato	Early blight	*Alternaria solani*	High N supply reduces disease severity	Blachinski et al., 1996
		Gray mold	*Botrytis cinerea*	High N supply increases plant resistance	Hoffland et al., 1999
	Potato	Early blight	*Alternaria solani*	High N supply reduces disease severity	Blachinski et al., 1996
	Rice	Blast disease	*Magnaporthe grisea*	High N supply increases disease severity	Long et al., 2000
	Wheat	Stripe rust	*Puccinia striiformis f. sp. tritici*	N supply decreases the infection severity	Devadas et al., 2014
Potassium	Wheat	Leaf blight	*Pyrenophora tritici-repentis*	Increased K supply lowers the disease severity	Sharma et al., 2005
		Leaf rust	*Puccinia triticina*	Increased K supply lowers the disease severity	Sweeney et al., 2000
	Rice	Sheath blight	*Rhizoctonia solani*	Reduced disease severity with an increased supply of K	Schurt et al., 2015
	Soybean	Pod and stem blight	*Diaporthe sojae*	Low K supply increases disease susceptibility	Snyder and Ashlock, 1996
	Peanut	Tikka leaf spot	*Cercospora arachidicola and Cercospora personatum*	Reduced disease incidences with increased K supply	Sharma et al., 2005
Phosphorus	Cucumber	Powdery mildew	*Sphaerotheca fuliginea*	P application reduces the disease severity	Reuveni et al., 2000
	Rice	Bacterial leaf blight	*Xanthomonas oryzae* pv. *oryzae*	P application reduces the disease severity	Huber and Graham, 1999
	Wheat	Flag smut	*Urocystis agropyri*	Application of P may increase the severity of diseases	Huber, 1980
Calcium	Soybean	Phytophthora stem rot	*Phytophthora sojae*	Ca application decreases the disease severity	Sugimoto et al., 2011
	Crucifers	Club root disease	*Plasmodiophora brassicae*	Sufficient soil Ca status reduces the disease incidence	Campbell and Arthur, 1990
Sulfur	Grapes	Powdery mildew	*Uncinula necator*	S application reduces the disease severity	Kruse et al., 2007
	Oilseed rape	Leaf spot	*Pyrenopeziza brassicae*	S application reduces the disease severity	Bloem et al., 2004
Magnesium	Rice	Brown spot	*Bipolaris oryzae*	Mg application reduces the disease severity	Moreira et al., 2015
	Corn	Corn stunt disease	*Spiroplasma kunkelii*	Mg application reduces the disease severity	Oliveira et al., 2005
**Micronutrients**					
Boron	Crucifers	Club root disease	*Plasmodiophora brassicae*	B application reduces the disease severity	Graham and Webb, 1991
	Grapevines	Eutypa dieback	*Eutypa lata*	B application increases resistance to disease	Rolshausen and Gubler, 2005
	Tomato	Tomato mosaic virus	TMV	B application reduces the disease severity	Graham and Webb, 1991
Zinc	Wheat	Fusarium head blight	*Fusarium graminearum*	Zn application reduces the disease infection	Graham and Webb, 1991
	Banana	Banana wilt	*Fusarium oxysporum* f. sp. *cubense*	Zn application increases the resistance to disease	Streeter et al., 2001
Copper	Tomato	Bacterial canker	*Clavibacter michiganensis* subsp *michiganensis*	Cu application reduces the disease incidence	Bastas, 2014
	Wheat	Powdery mildew	*Blumeria graminis* f. sp. *tritici*	Cu application suppresses the disease	Graham, 1983
Manganese	Potato	Common scab	*Streptomyces scabies*	Mn application reduces the disease incidence	Keinath and Loria, 1996
	Bent grass	Take-all disease	*Gaeumannomyces graminis* var. *avenae*	Mn application increases the resistance to disease	Carrow et al., 2001
Iron	Apple	Apple canker	*Sphaeropsis malorum*	Fe application increases the disease resistance	Graham, 1983
	Banana	Banana anthracnose	*Colletotrichum musae*	Fe application reduces the disease severity	Graham and Webb, 1991
Silicon	Paddy	Blast	*Magnaporthe oryzae*	Si application increases the plant resistance	Sun et al., 2010
		Brown spot	*Bipolaris oryzae*	Si application increases the plant resistance	Dallagnol et al., 2014
	Turf grass	Powdery mildew	*Blumeria graminis*	Si application reduces the disease severity	Zhang et al., 2006

This review presents the recent advances bridging the implications of mineral nutrients in sustaining plant health, with a focus on nutrient signaling and disease resistance. In addition, an attempt has been made to unravel the linkage between plant macro/micronutrients involved in the disease onset and progression, thereby ensuring sustainable crop production.

## Macronutrients mediated plant disease management

### Nitrogen

In the context of plant disease management, nitrogen (N) is an essentially important macronutrient required for the normal growth and development of the plant (Scheible et al., 2004). N plays a prominent role in varying metabolic and physiological processes, such as photosynthesis, amino acid synthesis, respiration, and tricarboxylic acid (TCA) cycle (Foyer et al., 2011). The N availability can restrict pathogen growth by alleviation and deployment of different plant defense mechanisms, and the different forms of N (NH_4_^+^ and NO_3_ form) are reported to have diverse effects on plant disease resistance (Bolton and Thomma, 2008; Mur et al., 2017). Several instances have been reported wherein N fertilization increased the plant disease incidence, for example, downy mildew, powdery mildew, leaf rust, stem rot, and rice blast diseases (Ballini et al., 2013; Devadas et al., 2014; Huang et al., 2017) while contrary results have been reported for diseases, such as take-all, gray mold, and leaf spot (Krupinsky et al., 2007; Lecompte et al., 2010). The excessive use of N fertilization in plants promotes succulent tissue growth and alleviates apoplastic amino acid concentration along with improving the plant canopy, which ultimately favors the growth of pathogenic spores (Neumann et al., 2004; Dordas, 2008).

The impact of N limitation on *Pseudomonas syringae* pv. *syringae* B728a when studied through an extensive transcriptomic assessment revealed the prominence of virulence-associated features, such as swarming motility, type three secretion system (T3SS), and metabolic pathways involved in gamma-aminobutyric acid (GABA) and polyketide metabolism (Bolton and Thomma, 2008). N starvation studies confirm its importance in initiating pathogenesis by stimulating the pathogen effector genes, such as the hypersensitive response and pathogenicity (*hrp*), avirulence (*avr*), and hydrophobin MPG1 genes in *Magnaporthe oryzae* (Pérez-García et al., 2001) while opposite results were documented for effectors from *Magnaporthe oryzae* (Huang et al., 2017) and *Passalora fulva* (ex *Cladosporium fulvum*) (Thomma et al., 2005). Defense enzymes are also an important arsenal possessed by plants in fighting the invading pathogen and N is observed to be involved in the stimulation of these enzymes during the host-pathogen interaction (Ngadze et al., 2012). The genes encoding the key regulatory enzymes of the defense pathway, such as phenyl ammonia lyase (PAL), cinnamate-4-hydroxylase (C4H), and 4-coumarate: CoA ligase (4CL), are all upregulated by N deficiency (Camargo et al., 2014) while a reduction in PAL activity has been observed with N fertilization (Sun et al., 2018). However, the relationship between N fertilization and plant disease is still unclear, but the understanding of the fundamental mechanism is noteworthy in crop production.

### Phosphorus

Phosphorus (P) is thought to be the second most commonly applied nutrient after nitrogen in crops but its role in resistance is seemingly inconsistent and variable. P is a part of many cell organic + molecules, such as deoxyribonucleic acid (DNA), ribonucleic acid (RNA), adenosine triphosphate (ATP), and is also involved in many metabolic processes taking place both in the plant and in the pathogen. During pathogen infection, the extracellular ATP is also received as a damage-associated molecular pattern (DAMP) since it is sensed by the plant when cellular damage is caused during pathogen colonization (Tanaka et al., 2014), considering it as a signaling molecule for the defense response activation in the plant (Cao et al., 2014). In recent reports, the role of extracellular ATP has been also proposed in jasmonic acid (JA)-induced defense response through direct activation of JA signaling in the *Arabidopsis* plant (Tripathi et al., 2018; Jewell et al., 2019). The beneficial effects of P application are also observed in controlling seedling and fungal diseases wherein the prolific root growth enables the plant to escape the disease (Huber and Graham, 1999). Various researchers have shown the significant effect of P fertilization in managing Pythium root rot in wheat (Huber, 1980) and reducing bacterial leaf blight in rice, downy mildew, blue mold, leaf curl virus disease in tobacco, pod and stem blight in soybean, yellow dwarf virus disease in barley, brown stripe disease in sugarcane, and blast disease in rice (Potash and Phosphate Institute [PPI], 1988; Reuveni et al., 1998; Huber and Graham, 1999; Kirkegaard et al., 1999; Reuveni et al., 2000). Campos-Soriano et al. (2020) reported overexpression of miR399 resulting in high Pi content and enhanced susceptibility to infection by the rice blast fungus *Magnaporthe oryzae* due to high phosphate fertilization.

### Potassium

Potassium (K) is an essential nutrient and the most plentiful inorganic cation found in plants (Shabala and Pottosin, 2010). K plays essential roles in enzyme activation, protein synthesis, photosynthesis, osmoregulation, stomatal movement, energy transfer, phloem transport, cation-anion balance, stress resistance (Marschner, 2012) crop yield, and quality improvement (Marschner, 2012; Oosterhuis et al., 2014). The plants with K starvation symptoms are observed to be more susceptible to disease in comparison to those having adequate K supply. A reduction in the incidence of fungal diseases (70%), bacterial diseases (69%), viral diseases (41%), and nematodes (33%) due to the profound K use was reported by Perrenoud (1990). Though K fertilization decreased the disease incidence in most of the cases, contrary results were also reported in some instances thereby categorizing the K impact on plant disease as “increased,” “decreased,” and having “no effect” or “variable effect” (Prabhu et al., 2007). The increased susceptibility in strawberries grown under K concentration excess toward *Colletotrichum gloeosporioides* and resistance alleviation in K fertilization absence due to starvation-induced synthesis of ROS and phytohormones were reported by Nam et al. (2006) which lead to enhanced plant stress tolerance (Amtmann et al., 2008). The increased K^+^ concentrations also decrease the prevailing intra-plant pathogen competition for nutrients (Holzmueller et al., 2007) and thereby enabling the plant to divert more resources to build the physical defense barrier and damage repair (Mengel, 2001). K is also an important facet in regulating the plant enzyme function by regulating the plants’ metabolite pattern and eventually varying its metabolite concentrations (Marschner, 2012). The synthesis of high-molecular-weight compounds (such as proteins, starches, and cellulose) and phenol concentration was significantly increased in plants with an adequate supply of K, which depressed the concentrations of low-molecular-weight compounds (soluble sugars, organic acids, amino acids, and amides) essential for diseases development in plant tissues, thereby making the plant less prone toward disease incidence (Prasad et al., 2010).

### Calcium

Calcium is an essential element, serving as one of the cell wall and membrane constituents and thereby contributing to the cell structure along with upholding the physical barriers against invading pathogens (White and Broadley, 2003). Owing to its significance in the structural role, the plants showing Ca deficiency are observed to be more prone to disease infection, and element exogenous supply has been shown to alleviate the plant’s resistance response toward the pathogen. A reduction in the Ca concentration within the plant increases susceptibility toward the fungi preferentially invading the xylem tissue and dissolving the cell wall of the conducting vessels increases, leading to wilting of the plant (Hirschi, 2004). In addition, Ca also plays an important role in serving as a secondary messenger for a variety of metabolic processes carried out within the plant during biotic stresses (Lecourieux et al., 2002). The Ca^2+^ signal is observed to be one of the earliest responses in the basal defense response triggering the signaling cascade required for the pathogen-associated molecular patterns (PAMPs) or host-derived damage-associated molecular patterns (DAMPs) that are recognized by surface-localized pattern-recognition receptors (PRRs) eventually leading to PAMP-triggered immunity (PTI) (Dodds and Rathjen, 2010).

### Sulfur

Sulfur (S) is an essential plant macronutrient having a pivotal role in plant disease resistance. The sulfur-containing defense compounds (SDCs) play versatile roles both in pathogen perception and initiating signal transduction pathways that are interconnected with various defense processes regulated by plant hormones (salicylic acid, JA, and ethylene) and ROS (Kunstler et al., 2020). The sulfur-containing amino acid (SAA) cysteine acts as a precursor of a large number of biomolecules, having major roles in plant disease resistance. Cysteine mediates spore germination and mycelial growth inhibition in a concentration-dependent manner in *Phaeomoniella chlamydospora* and *Phaeoacremonium minimum*, the two main causal agents of grapevine trunk disease (Roblin et al., 2018). The other important SAA in plants playing a central role in different defense reactions to biotic stresses is methionine (Met). A drastic reduction in the disease severity of Met-treated susceptible pearl millet cultivar (*Pennisetum glaucum*) infected by *Sclerospora graminicola* was reported by Sarosh et al. (2005). The Met treatment induces the generation of hydrogen peroxide (H_2_O_2_), a key element in plant defense signaling, leading to an upregulation in different defense-related gene expressions in grapevine (*Vitis vinifera*) (Boubakri et al., 2013). Sulfur-containing secondary metabolites play an important role in plant disease resistance and based on their mode of action can be classified into phytoalexins and phytoanticipins (Nwachukwu et al., 2012). In sulfur-deficient plants, there is a general gene downregulation responsible for sulfur-containing secondary metabolites synthesis and therefore the biosynthesis of S-containing phytoalexin (Camalexin) is also inhibited. Elemental sulfur (S0) can also be regarded as the only inorganic phytoalexin in plants that is accumulated during the xylem-invading fungal infection and bacterial pathogens infection, and its accumulation is faster and greater in disease-resistant genotypes than in susceptible lines (Cooper and Williams, 2004). The reactive sulfur species (RSSs) also play an important role in defense metabolism due to their participation in cellular signaling and regulatory processes. Two RSSs, hydrogen sulfide and sodium sulfite, have been shown to play important roles in plant disease resistance (Gao et al., 2012; Chen et al., 2014).

### Magnesium

Magnesium (Mg) is a vital cation, which influences an array of *in planta* physiological functions when the plant presents deficient or excess concentrations (Wang et al., 2020). It can also affect the pathogen invasion way into a plant by colonizing the plant phloem tissues, as it is present within the young phloem tissues under high Mg concentration and outside the cells under Mg deficit conditions. A low Mg concentration was detected in maize plants infected with corn stunt spiroplasma, which occurs due to the competition for Mg between the plant and the pathogen, thereby causing pronounced symptoms in the plant deficient in Mg (Nome et al., 2009). Mg deficiency during plant growth can also reduce the structural integration within the middle lamella and may also lower the energy production necessary for defense functions eventually leading to pathogen metabolites inactivation. A nutrient-rich environment favoring several phytopathogens occurs in the leaf tissue under the Mg deficiency condition due to sucrose and starch deposition in the leaf tissue (Huber and Jones, 2013). A higher clubroot disease incidence was also reported in soils showing lower Mg concentrations (Young et al., 1991). A drastic increase in the rate of disease infection and severity of peanut leaf spots caused by *Mycosphaerella arachidicola* was observed during the Mg deficient conditions (Bledsoe et al., 1945). An increase in pepper and tomato bacterial spot disease incidence caused by *Xanthomonas campestris* pv. *vesicatoria* was observed due to alleviated Mg levels (Woltz and Jones, 1979).

## Micronutrients mediated plant disease management

### Boron

Boron (B) is one of the least understood micronutrients showing widespread deficiency in plants around the globe (Brown et al., 2002). B nutrition-mediated physiological and metabolic activities that reduce disease susceptibility in the plant system are attributed to (1) strengthening cell wall structure through the formation of carbohydrate-borate complexes, which control carbohydrate transport and cell wall protein metabolism, (2) controlling cell membrane permeability and stability function, and (3) phenolics or lignin metabolism (Brown et al., 2002). In B deficient conditions, plant cell walls tend to swell and split, resulting in weakened intercellular space, which eventually weakens the physical barrier to the initial infection (Blevins and Lukaszewski, 1998). Sanjeev and Eswaran (2008) observed that B nutrition contributed to the maximum fungal mycelial growth inhibition and it can be used as a prokaryotic inhibitor at a certain concentration. The response of soil-borne phytopathogenic prokaryotes, such as *Ralstonia*, *Pectobacterium*, and *Pantoea*, to B can be assessed, and if boron concentration is not toxic to other beneficial plant-associated microorganisms, then altered B nutrition can be used as disease management effective means against the soil-borne plant pathogens.

### Zinc

Zinc (Zn), one of the crucial micronutrients, plays its role in plant response toward phytopathogens primarily activating or stabilizing metalloenzymes (Fones and Preston, 2012). Generally, the Zn deficient plants are more prone to pathogen attack (Streeter et al., 2001), thereby providing Zn the status of a significant element deciding the outcome of the plant-pathogen interaction. This results in limiting the invader’s entry or evading plant defense responses. Several studies suggest Zn fertilization role in reducing plant symptoms (Li et al., 2016; Machado et al., 2018); however, an increased susceptibility toward other pathogens was also reported due to protective Zn concentrations used against certain pathogens of the same host (Helfenstein et al., 2015). The studies conducted on the potential relationship between Zn availability status and fungal disease severity have reported an alleviated disease response in plants supplemented with Zn (Huber and Haneklaus, 2007; Khoshgoftarmanesh et al., 2010) while the contrary results were observed in soybean plants with varied Zn treatment, wherein either normal or high Zn fertilization had fewer positive counts for bacterial pustules caused by *Xanthomonas axonopodis* pv. *glycines* (Helfenstein et al., 2015). An evolutionary-conserved Zn-sensing phenomenon connecting root growth to pathogen response mechanism was stated by Bouain et al. (2018). In this study, the authors found that azelaic acid triggered by Azelaic Acid Induced1 (AZI1), belonging to the lipid transfer protein family (LTP) of the pathogenesis-related (PR) protein during systemic acquired resistance (SAR), regulated the plant growth and immunity responses on the basis of Zn availability status in plants.

### Copper

Copper (Cu) is one of the significant micronutrients required by plants that acts as a cofactor for several enzymes involved in respiration and electron transport proteins (Sommer, 1931). Cu is a plant protection essential part of controlling oomycetes, fungi, and bacteria for over a century. Although diseases can be managed by Cu applications, the lack of curative or systemic activity leads to Cu spray applications year after year (Graham et al., 2011). Plants with low Cu content show an increased disease incidence as a result of reduced lignification (Marschner, 1995). Cu fertilization in plants reduces the severity of fungal and bacterial diseases associated with cell wall stability and lignification (Broadley et al., 2012). The best evidence of a Cu effect on host plant resistance to disease can be observed in cases where Cu is applied in soil, and it lowers the leaf infection as evident in powdery mildew in wheat and ergot (*Claviceps* sp.) (Evans et al., 2007). The Cu synergistic effects can also be stated when it was used with other fungicides, such as Mancozeb, which leads to a reduction in canker and fruit spotting symptoms (Shoemaker, 1992). Cu compounds and their different combinations, in different studies, are reported to reduce sheath blight severity (*Rhizoctonia solani*) in rice (Khaing et al., 2014) and bacterial canker (*Clavibacter michiganensis* subsp. *michiganensis*) in tomatoes (Bastas, 2014).

### Manganese

Manganese (Mn) is an important micronutrient known for its efficacy on pathogen and resistance development in plants (Huber and Graham, 1999) owing to its ability for phenolic and lignin compound synthesis (Broadley et al., 2012). Cacique et al. (2012) reported that high Mn concentration on leaf tissues was found to decrease blast symptoms by *Pyricularia oryzae* in rice. Heine et al. (2011) observed that Mn can also contribute to black leaf mold disease control (*Pseudocercospora fuligena*) in tomatoes. Plants with inadequate Mn nutrition are observed to be unable in restricting the fungal hyphae penetration into the root tissues (Graham and Webb, 1991) while plants with adequate Mn nutrition show an alleviation in lignification and a reduction in aminopeptidase and pectin methyl esterase synthesis that is required essentially for fungal growth and for host cell wall breakdown, respectively (Carrow et al., 2001).

### Iron

Iron (Fe) is an essential micronutrient required by plants and pathogens having both positive and negative effects on the host and host disease resistance (Kieu et al., 2012; Aznar et al., 2015). Fe catalyzes ROS production that is used by the plant for alleviating the local oxidative stress as a defense response against the pathogens, thereby making iron play an intricate role in plant-pathogen interaction (Aznar et al., 2015). Fe fertilization is evident to be effective in antimicrobial compound synthesis leading to an indirect effect on the plant’s metabolic activity (Aznar et al., 2015). A reduction in symptom severity and pectate lyase encoding gene expression of the two soft rot-causing pathogens, such as *Dickeya dadantii* and *Botrytis cinerea*, was observed in plants showing Fe starvation (Kieu et al., 2012). Fe is also reported to enhance the fungal growth in certain plant-fungus interactions, as it was observed in *Phytophthora parasitica* var. *nicotianae*, wherein the fungal growth was observed to enhance in Fe^3+^ supplemented synthetic glucose asparagine medium (Hendrix et al., 1969). Fe also plays a potent role in *Pseudomonads* that are adapted to produce iron-chelating agents called siderophores in Fe-deficient soils, which, in turn, suppress certain fungal pathogens by starving them of iron (Calvent et al., 2001). Siderophores are also involved in some volatile antibiotic compounds’ synthesis (Thomashow, 1996). Depending on the host, the defense activation mechanism involves either their Fe scavenging property or receptor-mediated recognition as in the case of pattern-triggered immunity (Aznar and Dellagi, 2015). The reduced iron availability for fusaria-related wilts induced by fluorescent pseudomonads producing siderophores is reported as the main mechanism to reduce disease incidence in fusarium wilt of tomatoes (De Weger et al., 1986; Alabouvette, 1999; Hussain et al., 2016). In fact, soil suppressiveness to fusarium wilt of tomatoes has been mainly ascribed to Fe competition between the pathogenic *Fusarium oxysporum* isolates from the rhizosphere with the wild populations of fluorescent pseudomonads (Haas and Défago, 2005; Lemanceau and Alabouvette, 2008).

### Silicon

Silicon (Si) is not essentially a micronutrient but stands out eminently in its potential for decreasing several pathogens’ severity in varied crops belonging to the families *Poaceae*, *Equisetaceae*, and *Cyperaceae* (Huber et al., 2012; Pozza et al., 2015). The increased Si supply strongly reduces the number of lesions on young leaves, indicating an increase in disease resistance (Osuna-Canizalez et al., 1991). The silicates are known for inducing defense responses in plants by involving cell wall strengthening through alleviated phytoalexin production, increased lignification, PR protein synthesis, and phenolics production (Fawe et al., 2001; Oliveira et al., 2012).

Silicon is accumulated mainly in epidermal cells and exclusively on endodermal cells in roots and creates a physical barrier for fungal hyphae penetration into plant roots (Najihah et al., 2015). An increase in the activity of antioxidative enzymes (peroxidase, polyphenol oxidase, phenylalanine ammonia lyase, and lipoxygenase) was also observed after Si application (Shetty et al., 2011), which are considered the second line of defense for the pathogen entry into the host (Pozza et al., 2015). A significant reduction in lesion length of bacterial leaf blight (*Xanthomonas oryzae* pv. *oryzae*) among four rice cultivars was reported by Chang et al. (2002) following Si application, which was correlated with the soluble sugar content reduction in plant leaves amended with Si. Reduced severity in rice sheath blight disease was attributed to the increased lignin content and enhanced activities of antioxidative enzymes in rice leaves with Si addition. Therefore, knowing its effects on disease reduction, it can be included as an important component of crop protection.

## Conclusion

Since sustainable agriculture that uses increasing amounts of bio-fertilizers and organic amendments from a wide range of organic wastes represents a very important plant mineral nutrient source, it is fundamental to know the mechanisms of action by which such minerals can be involved in plant defense in several pathosystems. It is a general assumption that balanced nutrition leads to a healthy plant, which reduces disease susceptibility and infection. Thus, it is important to provide balanced nutrition at the due time when the nutrient can be most effectively used for disease control. Nutrient manipulation achieved by either modifying the nutrient availability or modifying the nutrient uptake for disease management or suppression has been reported in several studies. Fertilizers’ application affects plant disease development under field conditions either directly through the plant’s nutritional status or indirectly by affecting the conditions, which can influence the disease development, such as dense stands, changes in light interception, and humidity within the crop stand. It is a general assumption that balanced nutrition leads to a healthy plant, which reduces disease susceptibility and infection. Thus, it is important to provide balanced nutrition at the due time when the nutrient can be most effectively used for disease control.

## Author contributions

KPS provided the concept for the manuscript. RTr, RTe, and CK drafted the original manuscript. UDC, ES, TM, and AKS edited and supervised the final manuscript. All authors contributed to the article and approved the submitted version.
